# Supplementary figures and images for: The impact of preservation solutions for static cold storage on kidney transplantation outcomes: Results of a Brazilian nationwide multicenter study
Source: PLoS One. 2024 Jul 5;19(7):e0306056. doi: 10.1371/journal.pone.0306056 (PMC11226083; doi:10.1371/journal.pone.0306056)

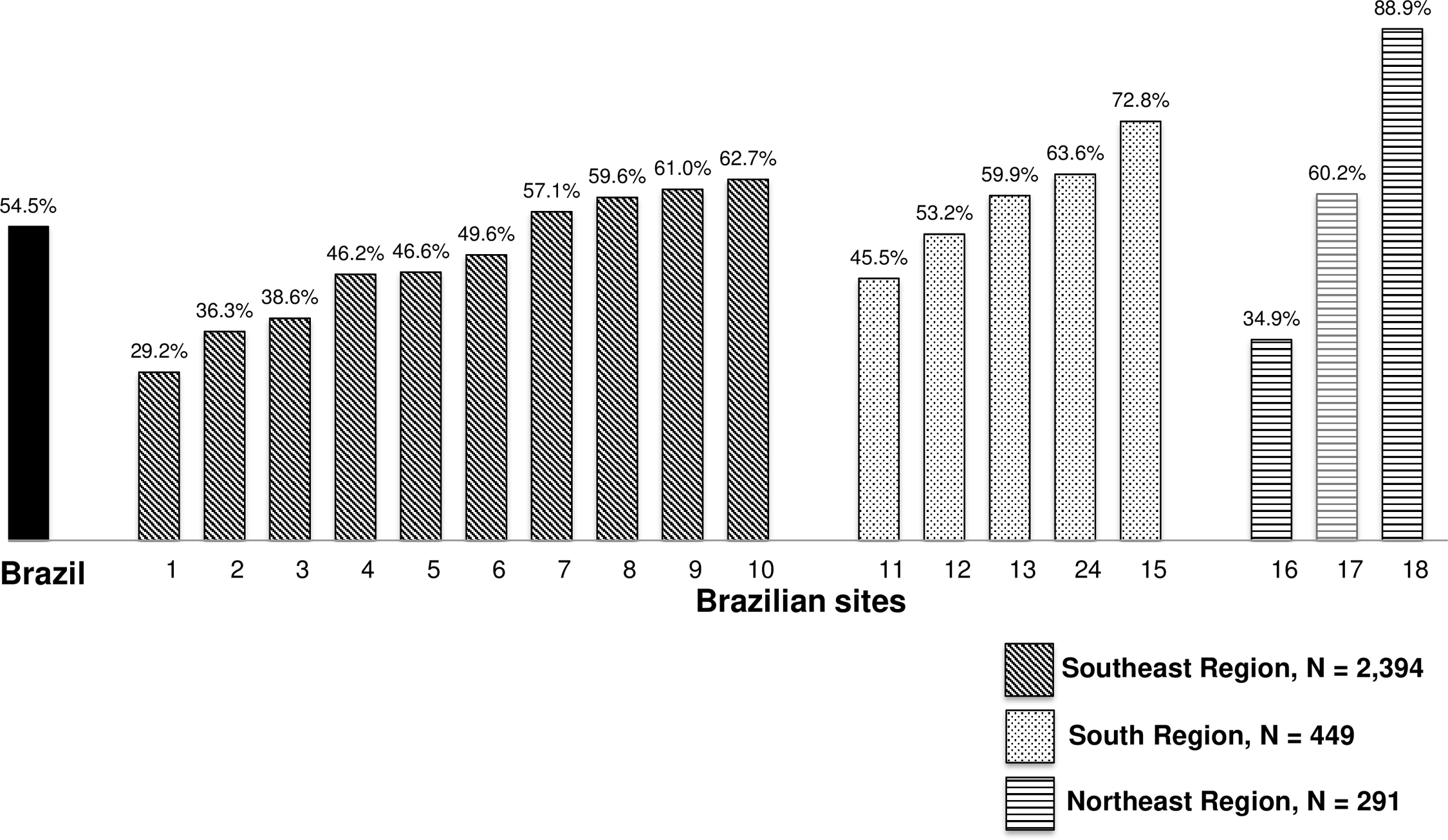

Supplement: S1 Fig — (TIF) [file pone.0306056.s001.tif]
